# Superpulsed Low-Level Laser Therapy Protects Skeletal Muscle of *mdx* Mice against Damage, Inflammation and Morphological Changes Delaying Dystrophy Progression

**DOI:** 10.1371/journal.pone.0089453

**Published:** 2014-03-05

**Authors:** Ernesto Cesar Pinto Leal-Junior, Patrícia de Almeida, Shaiane Silva Tomazoni, Paulo de Tarso Camillo de Carvalho, Rodrigo Álvaro Brandão Lopes-Martins, Lucio Frigo, Jon Joensen, Mark I. Johnson, Jan Magnus Bjordal

**Affiliations:** 1 Postgraduate Program in Rehabilitation Sciences, Universidade Nove de Julho (UNINOVE), São Paulo, SP, Brazil; 2 Postgraduate Program in Biophotonics Applied to Health Sciences, Universidade Nove de Julho (UNINOVE), São Paulo, SP, Brazil; 3 Department of Pharmacology, University of São Paulo, São Paulo, SP, Brazil; 4 Biological Sciences and Health Center, Cruzeiro do Sul University, São Paulo, SP, Brazil; 5 Department of Physiotherapy, Occupational Therapy and Radiography, Bergen University College, Bergen, Norway; 6 Faculty of Health and Social Sciences, Leeds Metropolitan University, Leeds, United Kingdom; 7 Physiotherapy Research Group, University of Bergen, Bergen, Norway; Stem Cell Research Institute, Belgium

## Abstract

**Aim:**

To evaluate the effects of preventive treatment with low-level laser therapy (LLLT) on progression of dystrophy in *mdx* mice.

**Methods:**

Ten animals were randomly divided into 2 experimental groups treated with superpulsed LLLT (904 nm, 15 mW, 700 Hz, 1 J) or placebo-LLLT at one point overlying the tibialis anterior muscle (bilaterally) 5 times per week for 14 weeks (from 6^th^ to 20^th^ week of age). Morphological changes, creatine kinase (CK) activity and mRNA gene expression were assessed in animals at 20^th^ week of age.

**Results:**

Animals treated with LLLT showed very few morphological changes in skeletal muscle, with less atrophy and fibrosis than animals treated with placebo-LLLT. CK was significantly lower (p = 0.0203) in animals treated with LLLT (864.70 U.l^−1^, SEM 226.10) than placebo (1708.00 U.l^−1^, SEM 184.60). mRNA gene expression of inflammatory markers was significantly decreased by treatment with LLLT (p<0.05): TNF-α (placebo-control = 0.51 µg/µl [SEM 0.12], - LLLT = 0.048 µg/µl [SEM 0.01]), IL-1β (placebo-control = 2.292 µg/µl [SEM 0.74], - LLLT = 0.12 µg/µl [SEM 0.03]), IL-6 (placebo-control = 3.946 µg/µl [SEM 0.98], - LLLT = 0.854 µg/µl [SEM 0.33]), IL-10 (placebo-control = 1.116 µg/µl [SEM 0.22], - LLLT = 0.352 µg/µl [SEM 0.15]), and COX-2 (placebo-control = 4.984 µg/µl [SEM 1.18], LLLT = 1.470 µg/µl [SEM 0.73]).

**Conclusion:**

Irradiation of superpulsed LLLT on successive days five times per week for 14 weeks decreased morphological changes, skeletal muscle damage and inflammation in *mdx* mice. This indicates that LLLT has potential to decrease progression of Duchenne muscular dystrophy.

## Introduction

Duchenne muscular dystrophy (DMD) is a recessive X-linked variety of muscular dystrophy affecting one in every 3500 males [1]–[3]. DMD is caused by mutation of the dystrophin gene at Xp21 resulting in an absence of the protein dystrophin. Symptoms usually arise between 3 to 5 years of age, and include calf muscle pseudohypertrophy, weakness of the proximal muscles (especially lower limbs) and abnormal gait. As the disease progresses through teenage years respiratory complications may appear in addition to headaches, nausea and fatigue [2]–[4]. Most individuals with DMD die by 30 years of age due these complications [3], [5]. There is no cure for the disease so the goal of treatment is to control symptoms to improve quality of life.

The most widely used preclinical model to study the degeneration and regeneration of muscle in DMD is the *mdx* (C57BL/10ScSn-*Dmd^mdx^*/J) mouse model. *Mdx* mice have a point mutation within the dystrophin gene preventing the expression and synthesis of dystrophin resulting in dystrophic-like symptoms similar to that seen in humans [3], [6], [7]. In early life (3 weeks) *mdx* mice present with periods of skeletal muscle degeneration and regeneration and as the animal ages (8 weeks) muscle atrophy and fibrosis develop [8]. Muscle fiber degeneration is accompanied by inflammatory and immune responses with death occurring prematurely in most mice at 24 months [9].

Inflammatory and immune responses have a critical role in the pathogenesis of DMD [9] so anti-inflammatory glucocorticoids (corticosteroids) are used as mainstay pharmacological treatment although they often lead to severe side-effects in the long-term and are often abandoned.

The term LASER means Light Amplification by Stimulated Emission of Radiation. Laser was developed at 1960s, and is light with special proprieties including monochromaticity and low divergence. Low-level laser therapy (LLLT) is the application of light for therapeutic purposes usually using a class 3B laser device with a mean output range of 10 mW–500 mW. There is strong evidence that LLLT promotes tissue regeneration, reduces inflammation and relieves pain [10]–[12]. The light used in LLLT is typically of narrow spectral width and in the red or near infrared (NIR) spectrum (600 nm–1000 nm), with a power density (irradiance) between 1 mW to 5 W/cm^2^ [13].

The first placebo-controlled clinical LLLT trial in musculoskeletal pain was published in 1980 and found that LLLT improved erythema, pain and grip strength in patients with rheumatoid arthritis of the hands [14]. Since then there has been a steady growth of evidence of efficacy for the management of various conditions including osteoarthritis [15] tendinopathies [10], [16], wounds [17], [18], back pain [19], neck pain [12], [20], peripheral nerve injuries [21] and stroke [22]. A major advantage of LLLT over pharmacological management is that there are minimal side-effects.

The use of LLLT to manage skeletal muscle fatigue and facilitate skeletal muscle recovery is a novel area of research. Recent studies performed by our research group have shown that when LLLT and light emitting diode therapy (LEDT) are applied before exercise the onset of skeletal muscle fatigue is delayed in both animals and humans and the status of biochemical markers related to skeletal muscle recovery is improved [23]–[32]. These findings suggest that LLLT may have protective effects on skeletal muscle tissue.

Evidence suggests that LLLT has physiological effects that may influence soft tissue metabolism in various pathologies including increased microcirculation [33], enhanced ATP synthesis and stimulating of mitochondrial respiratory chain [34] and mitochondrial function [35]. There are also reports that LLLT reduces the release of reactive oxygen species (ROS) and the activity of creatine kinase (CK, also known as creatine phosphokinase), and increases the production of antioxidants and heat shock proteins [36], [37].

With this perspective in mind, the aim of this study was to evaluate effects of preventive treatment with LLLT on progression of dystrophy in *mdx* mice, assessing skeletal muscle morphology, skeletal muscle damage and inflammation.

## Materials and Methods

### Animals

The experiments were started with 6 weeks old male *mdx* mice, housed in central animal house of Nove de Julho University with a 12-hours light/dark cycle and food and water *ad libitum*. Animals were purchased from the central animal house of Federal University of São Paulo (UNIFESP), Brazil. The study was conducted in accordance with policies and procedures of Brazilian laws and the Department of Health and Human Services in the USA. The experimental protocol was submitted and approved by the Animal Research and Care Committee of the Nove de Julho University, Sao Paulo, Brazil.

### Experimental groups

Ten animals were randomly divided into 2 experimental groups with 5 animals in each group:

- Placebo-control group: animals were treated with placebo LLLT (using a placebo laser probe) over the tibialis anterior muscle (bilaterally) for 5 times per week (Monday to Friday) for 14 weeks.
- LLLT group: animals were treated with active LLLT over the tibialis anterior muscle (bilaterally) for 5 times per week (Monday to Friday) for 14 weeks.

Animals were sacrificed at 20 weeks of age with an overdose of halothane 24 hours after the last LLLT treatment. After the removal of skin and connective tissue, tibialis anterior muscles were removed and processed for further analysis. Blood samples for creatine kinase (CK) analysis were collected by a single heart puncture.

### Procedures

#### Superpulsed LLLT treatment

A GaAs diode superpulsed laser with a frequency of 700 Hz, mean output power of 15 mW and wavelength of 904 nm (infrared) was used. A complete description of LLLT parameters is presented in table 1.

**Table 1:** Superpulsed LLLT parameters.

	Placebo-control group	LLLT group
**Wavelength:**	904 nm	904 nm
**Peak power:**	0 W	10.4 W
**Pulse width:**	-	180 ns
**Pulse frequency:**	-	700 Hz
**Average power:**	0 mW	15 mW
**Illuminated area per hind limb:**	0.2 cm^2^	0.2 cm^2^
**Peak irradiance:**	0 W/cm^2^	52 W/cm^2^
**Average irradiance:**	0 mW/cm^2^	75 mW/cm^2^
**Time:**	67 s per hind limb	67 s per hind limb
**Energy:**	0 J	1.005 J
**Fluence:**	0 J/cm^2^	5.025 J/cm^2^

**Figure 1:**
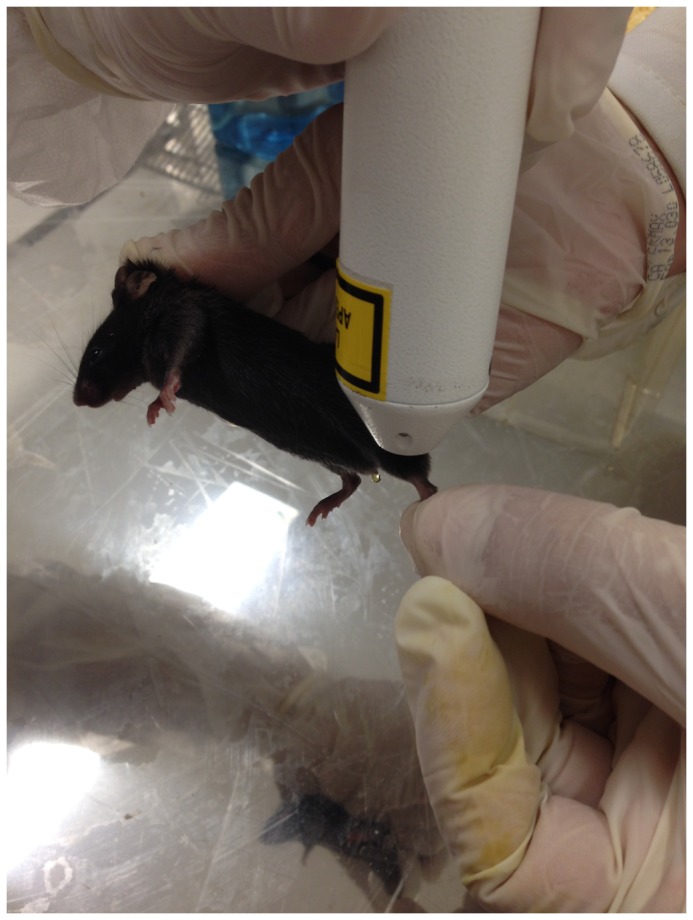
LLLT irradiation being performed to *mdx* mice.

The optical power of the laser device was calibrated with a Newport multifunction optical meter model 1835C, before, during and after the experiment. The stability of the laser output during laser irradiation was measured by collecting light with a partial reflect (4%). The spot size was 0.2 cm^2^, and the laser illumination spot was placed in direct contact with the skin (shaved) overlying the central part of tibialis anterior muscle. Animals received irradiation at this single point (bilaterally) 5 times per week (Monday to Friday) for 14 weeks. Irradiation lasted 67 s, with a fixed power density of 75 mW/cm^2^. The total delivered energy was 1.0 J per session for the LLLT group. The placebo-control group was treated in an identical manner using an identical laser probe that delivered 0 mW of output power (i.e. a placebo laser probe). The therapist who performed the treatment was blinded to group allocation. The energy dose chosen was based on two previous studies performed by our research group using identical LLLT parameters that found that LLLT protected skeletal muscle tissue by delaying skeletal muscle fatigue and improving biochemical markers of skeletal muscle damage in rats [28], [30]. Figure 1 illustrates the laser irradiation being performed.

### Outcomes

Analyses of histology, blood and PCR of biochemical markers were performed by a blinded observer.

#### Histology

Muscle tissue samples were fixed in a 10% formalin solution for 72 hours and then dehydrated in a series of alcohol baths beginning with 50% and progressing to 100% (SYNTH) and cleared in xylol for 4 hours (SYNTH). The samples were placed in adequate aluminum recipients with Paraplast for 4 hours for impregnation and then placed in a small recipient, covered with paraffin and left to harden, forming a block containing the tissue sample. Slices measuring 5 µm in thickness were cut on a microtome (LEICA RM 2125 RT), washed and placed in a water bath. The cuts were stained with hematoxylin and eosin and mounted on permanent slides for subsequent analysis under an optical microscope (Nikon, Eclipse E-200 model, China). The specimens were photographed using a microphotographic camera (Dino-Lite Digital Microscope, DinoEye AM423X model, Brazil) connected to a microcomputer. Standardized photos were taken of all groups at magnifications of ×100 and ×400.

#### CK analysis

For the analysis of CK, 3 ml of blood were collected from each animal through heart puncture immediately prior to euthanasia. The material was centrifuged and the supernatant analyzed. CK was determined using the Labtest commercial kit (Brazil). One ml of the working reagent was pipetted; 0.02 ml of the serum sample was homogenized and immediately transferred to a cubette at 37°C for 2 minutes. The reading of the initial absorbance was performed using an enzyme-linked immunosorbent assay following the instructions of the commercial kit.

#### RNA isolation and real-time polymerase chain reaction (RT-PCR) analysis

Firstly, muscles were thawed, and Trizol was immediately added (Gibco BRL, Life Technologies, Rockville, MD, USA, 1 ml/100 mg tissue). Then, muscles were homogenized for the recovery of total RNA, according to the manufacturer's instruction.

DNase I was employed to digest DNA to obtain RNA purification and the integrity of RNA was verified by agarose gel electrophoresis. Total RNA (2 µg) was used for first-strand cDNA synthesis [reverse transcriptase (RT)] using SuperScript II. In addition, RNaseOUT was also added to protect the RNA during this process. Three pooled RNA aliquots were routinely sham reverse transcribed (i.e. reverse transcriptase omitted) to ensure the absence of DNA contaminants. Diluted RT samples (1∶10) were submitted to Real Time PCR amplification using Platinum Sybr QPCR Supermix-UDG and specific oligonucleotides (designed using http://www.ncbi.nlm.nih.gov/tools/primer-blast/). The primers used were: TNF-α (forward: CCACCACGCTCTTCTGTCTA; reverse: AGGGTCTGGGCCATAGAACT), IL-1β (forward: TTGACGGACCCCAAAAGATG; reverse: AGAAGGTGCTCATGTCCTCAT), IL-6 (forward: GAGCCCACCAAGAACGATAG; reverse: TCAGTCCCAAGAAGGCAACT), IL-10 (forward: CAGCCGGGAAGACAATAACT; reverse: ATGTTGTCCAGCTGGTCCTT), COX-2 (forward: TGAGCACAGGATTTGACCAG; reverse: CCTTGAAGTGGGTCAGGATG), HPRT was used as an internal control (forward: TCCTCCTCAGACCGCTTT; reverse: TTTTCCAAATCCTCGGCATAATG).

The conditions for PCR were as follows: 50°C – 2 min; 95°C – 2 min, followed by 30 cycles of 95°C – 15 sec; 60°C – 1 min, followed by 72°C – 15 sec. Cycle threshold (Ct) values were recorded for each gene, and the results of genes of interest were normalized to results obtained with the internal control gene. Delta-Delta-Ct (ddCt) values were calculated and results expressed as fold increases. All oligonucleotides and reagents utilized were purchased from Invitrogen Co. (USA).

### Statistical Analysis

All data were normally distributed so statistical analysis was performed using two-tailed unpaired t-tests. The statistical level of significance was set at p<0.05.

## Results

**Figure 2:**
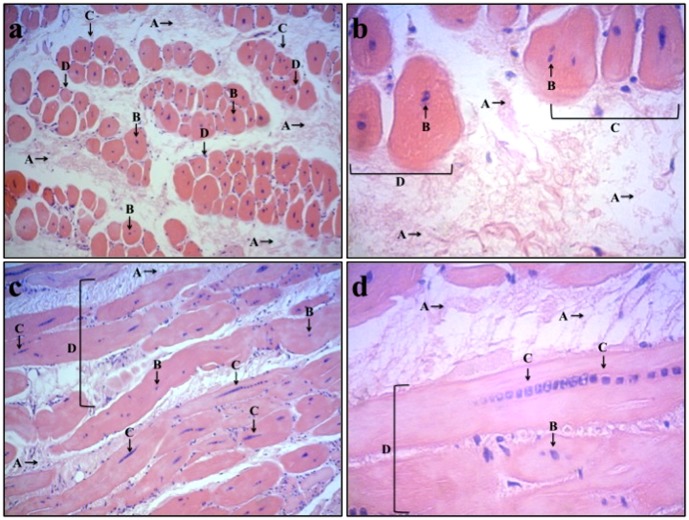
Photomicrograph of morphological aspects in muscle tissue of control group. Figure 1a (placebo-control group - transversal): large amount of fibrous tissue (A), nuclei dislocated to the center of muscle fibers (B), decreased number of muscle fibers (C), and decreased size of muscle fibers (D), Magnification: ×100. Figure 1b (placebo-control group - transversal): large amount of fibrous tissue (A), nuclei dislocated to center of muscle fiber (B), decreased number of muscle fibers (C), and decreased size of muscle fibers (D), Magnification: ×400. Figure 1c (placebo-control group - longitudinal): large amount of fibrous tissue (A), nuclei dislocated to center of muscle fiber (B), clustering of nuclei in the center of muscle fiber (C), and decreased number of muscle fibers (D), Magnification: ×100. Figure 1d (placebo-control group - longitudinal): large amount of fibrous tissue (A), nuclei dislocated to center of muscle fiber (B), clustering of nuclei in the center of the muscle fibers (C), and decreased size of muscle fibers (D), Magnification: ×400.

**Figure 3:**
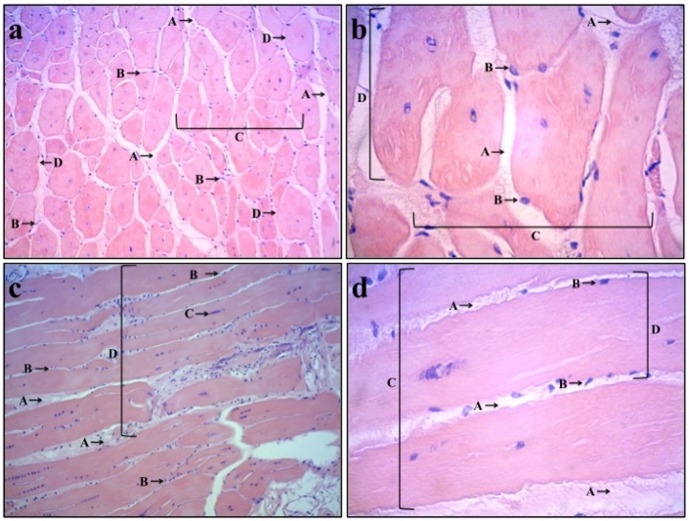
Photomicrograph of morphological aspects in muscle tissue of LLLT groups. Figure 2a (LLLT group - transversal): small amount of fibrous tissue (A), nuclei situated in the periphery of muscle fibers (B), majority of muscle fibers normal (C), and majority of muscle fibers of normal size (D), Magnification: ×100. Figure 2b (LLLT group - transversal): small amount of fibrous tissue (A), nuclei situated in the periphery of muscle fibers (B), normal number of muscle fibers (C), and majority of muscle fibers of normal size (D), Magnification: ×400. Figure 2c (LLLT group - longitudinal): small amount of fibrous tissue (A), nuclei situated in the periphery of muscle fibers (B), small clustering of nuclei in the center of muscle fiber (C), and normal number of muscle fibers (D), Magnification: ×100 Figure 2d (LLLT group - longitudinal): small amount of fibrous tissue (A), nuclei situated in the periphery of muscle fibers (B), normal number of muscle fibers (C), and increased size of muscle fibers (D), Magnification: ×400.

The analysis of animals in the placebo-control group showed extensive fibrosis, decreased number of muscle fibers, decreased size of muscle fibers and clustering of nuclei in the center of muscle fibers indicative of a degenerative process of muscle tissue. Animals in the LLLT group had limited fibrosis, normal number and size of muscle fibers and nuclei in the periphery of muscle fibers indicative of delayed progression of pathological changes in the skeletal muscle tissue. Morphological aspects of skeletal muscle tissue in placebo-control group and LLLT group are shown in figures 2 and 3.

**Figure 4:**
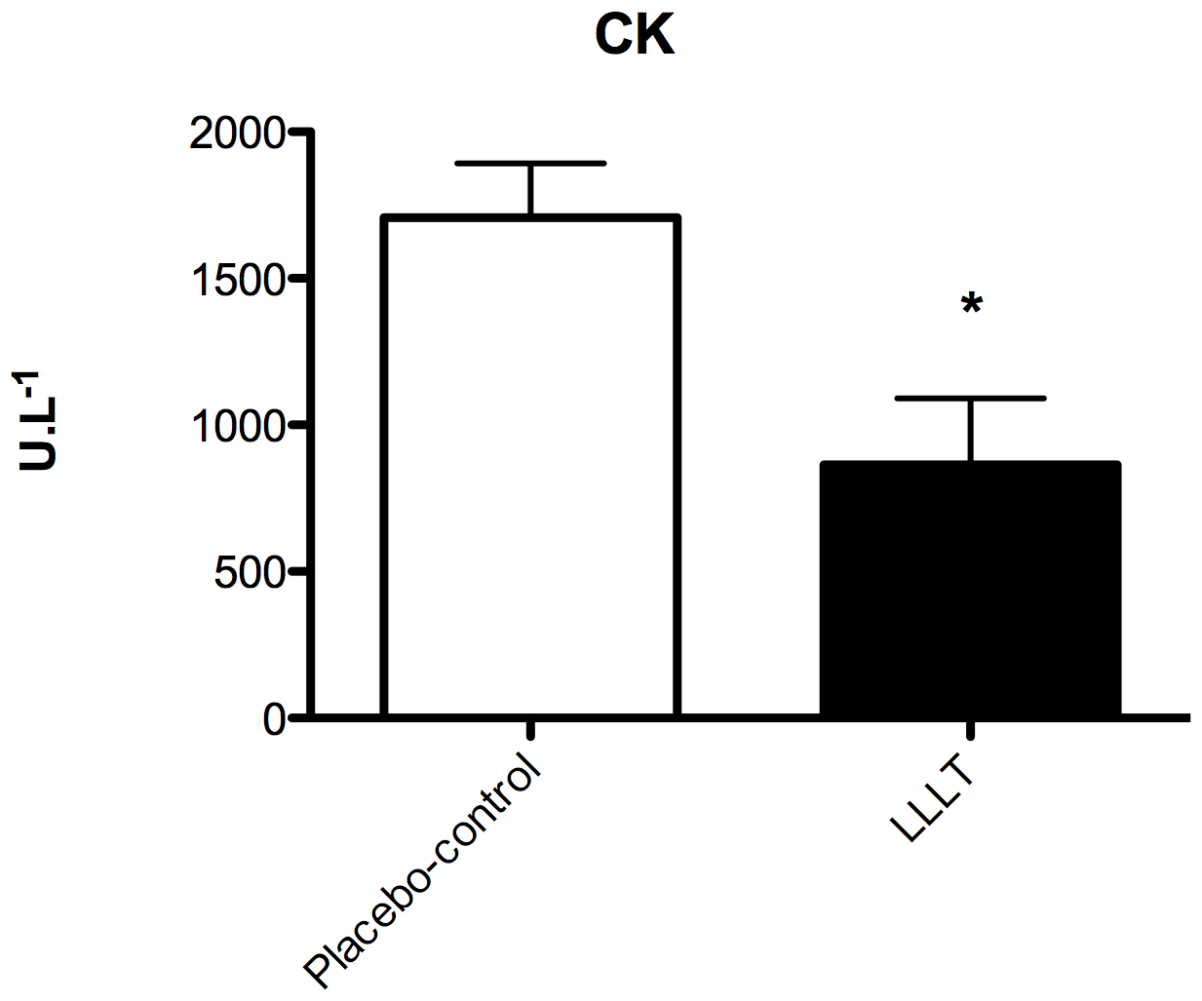
CK activity levels in the placebo-control group and in the LLLT group (n = 5 animals per group). The * indicates significant difference compared with placebo-control group (p = 0.0203). Error bars indicate SEM.

CK activity, indicative of muscle damage, was significantly lower (p = 0.0203) in the LLLT group (864.70 U.l^−1^, SEM 226.10) compared with the placebo-control group (1708.00 U.l^−1^, SEM 184.60, figure 4).

**Figure 5:**
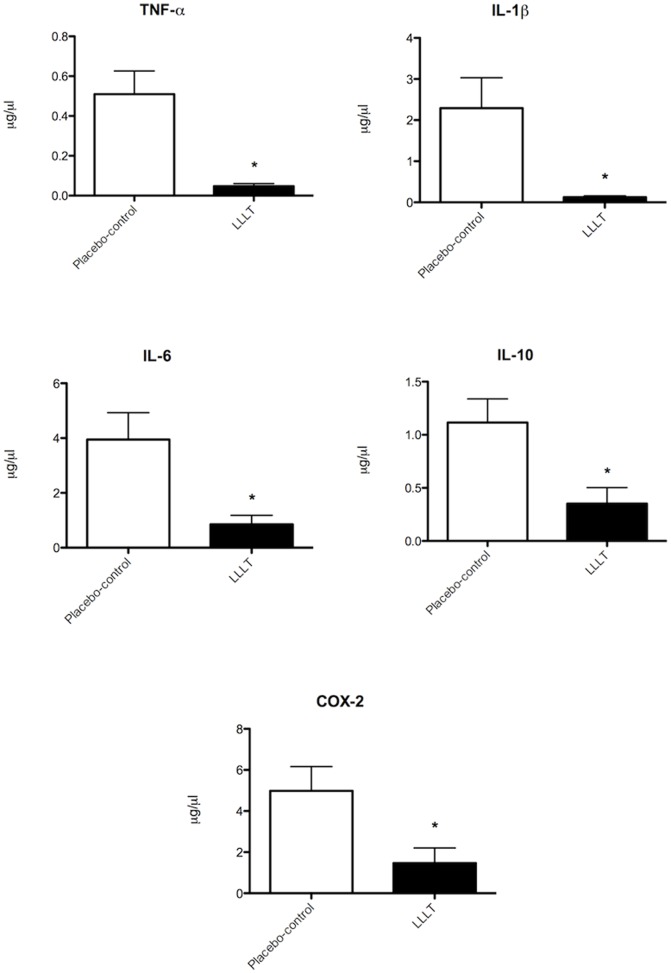
mRNA gene expression of TNF-α, IL-1β, IL-6, IL-10, and COX-2 in placebo-control group and LLLT group (n = 5 animals per group). The * indicates a significant difference compared with placebo-control group (p = 0.0042 for TNF-α, p = 0.0189 for IL-1β, p = 0.0174 for IL-6, p = 0.0218 for IL-10, and p = 0.0355 for COX-2, respectively). Error bars indicate SEM.

mRNA gene expression of inflammatory markers was significantly lower in the LLLT group compared with the placebo-control group. TNF-α gene expression levels were 0.51 µg/µl (SEM 0.12) in placebo-control group and 0.048 µg/µl (SEM 0.01) in LLLT group (p = 0.0042). IL-1β was 2.292 µg/µl (SEM 0.74) in the placebo-control group and 0.12 µg/µl (SEM 0.03) in the LLLT group (p = 0.0189). mRNA gene expression of IL-6 was 3.946 µg/µl (SEM 0.98) in the placebo-control group and 0.854 µg/µl (SEM 0.33) in the LLLT group (SEM p = 0.0174). IL-10 was 1.116 µg/µl (SEM 0.22) in the placebo-control group and 0.352 µg/µl (SEM 0.15) in the LLLT group (p = 0.0218). mRNA gene expression of COX-2 was 4.984 µg/µl (SEM 1.18) in the placebo-control group and 1.470 µg/µl (SEM 0.73) in the LLLT group (p = 0.0355). The results of mRNA gene expression of inflammatory markers are summarized in figures 5.

## Discussion

This study used gene-deficient *mdx* mice as an experimental model of DMD [38]. Fibrosis and inflammation have critical roles in the progression of DMD [9] and therefore treatment with glucocorticoid drugs which have anti-inflammatory actions is widely used [39]. Non-steroidal anti-inflammatory drugs (NSAIDs) are also a treatment option [40]. Longitudinal studies find that glucorticoids are beneficial in the management of DMD by prolonging self-ambulation, reducing the need for scoliosis surgery [41] and delaying the onset of cardiomyopathy [42]. However, high quality evidence from randomized controlled trials is lacking and glucocorticoids have side-effects on bone growth resulting in treatment having to be abandoned [39], [43]. Consequently alternative approaches to reduce inflammation, fibrosis and necrosis are being considered. Therapeutic targets include nuclear hormone receptors, calcium channels and NADPH-oxidases. Promising targets to counteract DMD progression also include strategies to inhibit nuclear factor-kappaB (NF-kappaB), transforming growth factor-alpha (TGF-alpha), transforming growth factor-beta (TGF-beta) and inhibiting the production or action myostatin [39].

To our knowledge this is the first study to investigate the effect of LLLT on the progression of DMD. Over the years LLLT has been used to treat a variety of inflammatory disorders like osteoarthritis [15], tendinopathies [10], [16] and acute cancer therapy-induced oral mucositis [44]. Several studies on animals and humans have shown that LLLT with both red and infrared wavelengths modulates the release of inflammatory markers including PGE_2_, TNF-α, IL-1β and plasminogen activator [45]. LLLT also modulates several aspects of the inflammatory process including oedema and hemorrhagic formation, necrosis, neutrophil cell influx and the activity of macrophages, lymphocytes and neutrophils [10], [46]–[49]. LLLT has been shown to inhibit the NF–Kappa signaling pathway [50] and to modulate expression of inducible nitric oxide synthase (iNOS) [51].

Our placebo-controlled study found that LLLT decreased mRNA gene expression of various inflammatory markers including TNF-α, IL-1β, IL-6, IL-10 and COX-2. This suggests that LLLT has protective effects on skeletal muscle tissue. Furthermore, CK activity was significantly lower in the LLLT group compared with the placebo-control group and this suggests that LLLT reduced the progression of muscle damage. Reduction of CK activity has also been reported after successful glucocorticoid therapy and is associated with clinical benefit [52].

Previous studies performed by our research group using rats have found that LLLT delivered using a 904 nm wavelength and a dose of 1 J irradiated before tetanic contractions significantly increased skeletal muscle performance [28], [30], and significantly decreased CK activity [28], [30] and COX-2 mRNA gene expression [30]. Recently, Hayworth et al. [53] found that a single dose of LLLT increased cytochrome c-oxidase activity in intact skeletal muscle tissue 24 hours after irradiation. Additionally, there was a dose and fiber type-dependent increase in cytochrome c-oxidase in skeletal muscle fibers suggesting that LLLT up-regulated mitochondrial activity increasing ATP production into muscle cells and decreasing oxidative stress and ROS production. These effects may contribute to the mechanism by which LLLT protects skeletal muscle against degeneration.

We recognize that our study evaluated morphological aspects of skeletal muscle, biochemical marker of damage and gene expression of inflammatory markers in an animal model, so we understand that this represents a limitation and we express caution at extrapolating our findings into humans at this stage. Nevertheless, LLLT has a strong safety profile and reports of side effects in an evidence base of over 200 randomized controlled clinical trials are few and minor. Therefore, we believe serious consideration should be given to the potential of LLLT as a treatment option of long-term conditions like DMD. Future studies would include investigation of the effects of LLLT on protein expression of inflammatory markers and functional aspects of DMD and the determination of optimal parameters to inform the design of robust clinical trials. We hope that our findings may initiate interest in the use of LLLT as a potentially useful adjunct for DMD.

## Conclusion

Superpulsed LLLT administered using a wavelength of 904 nm and dose of 1 J on successive days, 5 times per week for 14 weeks decreased morphological changes, skeletal muscle damage and inflammation in *mdx* mice. This suggests that LLLT may decrease progression of DMD. Further studies are needed to elucidate the mechanism of action, effects on functional outcomes and to establish optimal parameters of application to inform clinical use.
